# Calpain inhibitor MDL28170 improves the transplantation-mediated therapeutic effect of bone marrow-derived mesenchymal stem cells following traumatic brain injury

**DOI:** 10.1186/s13287-019-1210-4

**Published:** 2019-03-15

**Authors:** Jiangnan Hu, Lefu Chen, Xujun Huang, Ke Wu, Saidan Ding, Weikan Wang, Brian Wang, Charity Smith, Changhong Ren, Haoqi Ni, Qichuan ZhuGe, Jianjing Yang

**Affiliations:** 1 Zhejiang Provincial Key Laboratory of Aging and Neurological Disorder Research, The First Affiliated Hospital of Wenzhou Medical University, Wenzhou, 325000 China; 2 Department of Pharmaceutical Sciences, University of North Texas Health Science Center, Fort Worth, TX 76107 USA; 3 Department of Intensive Care Unit (ICU), Hengdian Wenrong Hospital, Jinhua, 322100 China; 4 Beijing Key Laboratory of Hypoxic Conditioning Translational Medicine, Xuanwu Hospital, Capital Medical University, Beijing, China

**Keywords:** Traumatic brain injury, Bone marrow-derived mesenchymal stem cells, Preconditioning, Calpain inhibitor, MDL28170, Transplantation

## Abstract

**Background:**

Studies have shown that transplantation of bone marrow-derived mesenchymal stem cells (BMSCs) protects against brain damage. However, the low survival number of transplanted BMSCs remains a pertinent challenge and can be attributed to the unfavorable microenvironment of the injured brain. It is well known that calpain activation plays a critical role in traumatic brain injury (TBI)-mediated inflammation and cell death; previous studies showed that inhibiting calpain activation is neuroprotective after TBI. Thus, we investigated whether preconditioning with the calpain inhibitor, MDL28170, could enhance the survival of BMSCs transplanted at 24 h post TBI to improve neurological function.

**Methods:**

TBI rat model was induced by the weight-drop method, using the gravitational forces of a free falling weight to produce a focal brain injury. MDL28170 was injected intracranially at the lesion site at 30 min post TBI, and the secretion levels of neuroinflammatory factors were assessed 24 h later. BMSCs labeled with green fluorescent protein (GFP) were locally administrated into the lesion site of TBI rat brains at 24 h post TBI. Immunofluorescence and histopathology were performed to evaluate the BMSC survival and the TBI lesion volume. Modified neurological severity scores were chosen to evaluate the functional recovery. The potential mechanisms by which MDL28170 is involved in the regulation of inflammation signaling pathway and cell apoptosis were determined by western blot and immunofluorescence staining.

**Results:**

Overall, we found that a single dose of MDL28170 at acute phase of TBI improved the microenvironment by inhibiting the inflammation, facilitated the survival of grafted GFP-BMSCs, and reduced the grafted cell apoptosis, leading to the reduction of lesion cavity. Furthermore, a significant neurological function improvement was observed when BMSCs were transplanted into a MDL28170-preconditioned TBI brains compared with the one without MDL28170-precondition group.

**Conclusions:**

Taken together, our data suggest that MDL28170 improves BMSC transplantation microenvironment and enhances the neurological function restoration after TBI via increased survival rate of BMSCs. We suggest that the calpain inhibitor, MDL28170, could be pursued as a new combination therapeutic strategy to advance the effects of transplanted BMSCs in cell-based regenerative medicine.

**Electronic supplementary material:**

The online version of this article (10.1186/s13287-019-1210-4) contains supplementary material, which is available to authorized users.

## Background

Traumatic brain injury (TBI) remains a major health problem worldwide. The pathophysiology of brain injury after head trauma is complicated and can be characterized by the initial injury and the subsequent injury that ensues days after the trauma [1]. The incidence of TBI is increasingly a major cause of morbidity and mortality among all traumas [2, 3], leading to considerable disability, mortality, and functional impairment that severely affects the quality of life [4, 5].

Currently, therapeutic strategies for TBI mainly include controlling the secondary damage through the administration of neurotrophic drugs and promoting rehabilitation training of neurological function [6]. However, these therapeutic effects were less than optimal and novel strategies remain to be found. In the last decade, several studies regarding bone marrow-derived mesenchymal stem cell (BMSC) transplantation as an alternative therapy for TBI [7–9] have shown great promise in animal experimental models [10–13] and in the clinic [14, 15]. The benefits of the transplanted BMSC are twofold: (i) its ability to commit to a neural lineage and migrate long distances to injury sites allows it to serve as a direct replacement for dead or dying cells [16, 17] and (ii) its presence at the lesion site indirectly influences the microenvironment through the secretion of growth factors, which rescues neuronal cells and promotes the proliferation of neuroblasts [18, 19]. Yet, the survival and viability of BMSCs are relatively poor in the injured brain, and the early death of transplanted cells limits the BMSC-based therapies [20, 21]. To exploit their full therapeutic potential, there is a critical need to determine the cause(s) of early death and develop strategies to enhance their survival.

Factors present at the lesion site can induce host tissue damage and contribute to the death of transplanted cells. Recent studies have demonstrated a pivotal role of calpain, a calcium-mediated cysteine protease, in mediating necrotic and apoptotic cell death [22]. The resultant proteolysis of cytoskeletal, membrane, and myelin proteins is strongly implicated in the secondary damage, which includes the death of motor neurons, axonal degeneration, oligodendrocyte death, and demyelination-associated with Ca^2+^ accumulation [23]. Meanwhile, the inflammatory response acts as a key step in the secondary injury cascade following TBI that also contributes to the death of transplanted cells. It is characterized by the recruitment of peripheral leukocytes into the cerebral parenchyma, activation of resident immune cells [24, 25], and initiation of the inflammatory cascade mediated by the release of pro- and anti-inflammatory cytokines [26, 27]. Several lines of evidence have highlighted calpain’s critical role in driving the inflammatory response, citing it as one of the earliest pro-inflammatory cytokines to be upregulated after neurotrauma [28–30].

Calpain modulates key processes that govern the pathogenesis of neurodegeneration and pro-inflammatory response [29, 31]. Therefore, calpain inhibitors can be presumed to be effective therapeutic agents for attenuating calpain’s actions [32, 33]. Here, we used the calpain inhibitor, MDL28170, which has the ability to cross the blood-brain barrier (BBB) and cell membranes. MDL28170 was reported to prevent the upregulation of pro-inflammatory factors induced by calpain [28, 34]. It also exerts neuroprotective effects in a variety of neurological injuries such as TBI, spinal cord injury, stroke, and Parkinson’s disease [31, 34, 35].

This study was designed to explore whether the anti-neurodegeneration and anti-inflammatory effects of the calpain inhibitor, MDL28170, could exert a certain protective effect against damage caused by TBI and enhance the survivability of grafted BMSCs in the contused rat brain to further improve the therapeutic effects of BMSC-based TBI therapy.

## Methods

### Experimental groups and TBI model

**Fig. 1:**
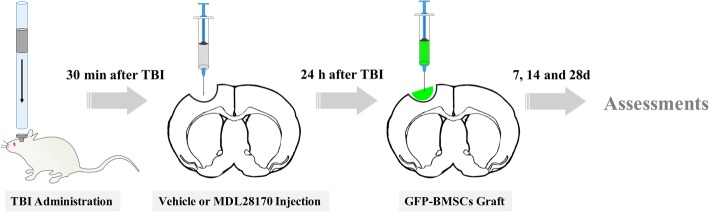
Schematic of experimental design. To induce traumatic brain injury, a 50-g circular hammer was allowed to fall through the guide stick from a height of 30 cm. Thirty minutes after injury, animals were administered the calpain inhibitor, MDL28170, intracranially at the site of the lesion. At 24 h after injection, animals were transplanted with GFP-BMSCs at the lesion cavity. Rats then underwent histological and neurological functional assessments at the different time points, 7, 14, and 28 days. TBI, traumatic brain injury; BMSCs, bone marrow mesenchymal stem cells

All protocols involving the use of animals followed the guidelines set out by the Animal Care Committee of Wenzhou Medical University (China). Male Sprague-Dawley (SD) rats weighing about 200–240 g were used in the study. The rats were randomly assigned to the following groups: a sham-operated group, a TBI group (rats subjected to TBI only), a vehicle-treated group (rats subjected to TBI and received vehicle treatment at 30-min post-injury, namely, 20% dimethylsulfoxide (DMSO) in normal saline, *v*/*v*), a MDL28170 treatment group (rats subjected to TBI and received MDL28170 treatment at 30-min post-injury), a green fluorescent protein (GFP)-BMSCs group (rats subjected to TBI and received GFP-BMSC transplantation at 24-h post-injury), and a MDL28170+GFP-BMSC transplantation group (rats subjected to TBI and received MDL28170 treatment at 30 min post-injury, then transplanted with GFP-BMSCs at 24 h post-injury) (Fig. 1).

For the TBI model, rats were anesthetized by intraperitoneal injection with 10% chloral hydrate (0.4 mL/100 g), shaved, and placed in a stereotaxic frame (Kopf Instruments, Tujunga, CA, USA). The scalp was incised at the midline, exposing the skull. A right parietal bone was drilled with a hole of 5 mm in diameter without damaging the dura mater. The center of the craniotomy was 3.5 mm posterior and 2.5 mm lateral to the bregma. The parietal contusion was produced by allowing a 50 g hammer to fall from a 30-cm guide stick. At the end of the procedure, the exposed dura was covered with bone wax and the scalp was sutured. Sham-operated rats were surgically treated with right parietal craniotomy but without injury to the brain. After trauma, the rats were placed in a warmed, oxygenated recovery chamber with free under controlled temperature (25 ± 0.5 °C) and humidity (55 ± 5%). Rats were housed under the 12/12-h light-dark cycle and had unlimited access to food and water. Postoperative care included injections of penicillin to prevent infection. Rats that lacked neurological deficits after TBI administration were excluded.

### MDL28170 administration

MDL28170 (carbobenzoxy-valyl-phenylalanine, calpain inhibitor I, readily crosses the blood-brain barrier and cell membranes; Cat. No. M6690, Sigma, St Louis, USA) [34] was first dissolved in dimethylsulfoxide (DMSO) and then diluted with 0.9% NaCl to a final concentration of 50 mM. Final concentration of DMSO was 20%, *v*/*v*. At 30 min post TBI, 1.0 μl of 50 mM MDL28170 was injected into the center of the lesion site at a depth of 1.0 mm using a microinjection needle clamped by a stereotaxic instrument. Controls received an equal volume of the vehicle (20% DMSO, *v*/*v*).

### Cell preparation, characterization and transplantation

Primary bone marrow stem cells were harvested from the bone marrow of SD rats, cultured as monolayer, then transfected with a lentiviral construct containing a green fluorescent protein (GFP) expression motif. GFP-BMSCs were cultured in a BMSC growth medium, passaged and amplified to the first generation, and frozen at − 80 °C. When needed, GFP-BMSCs were thawed and transferred to tubes containing the growth medium then centrifuged at 1000 rpm for 5 min. After removing the supernatant, cells were dispersed gently with 2–3 mL of medium. The cell suspension was transferred to a 25-cm^2^ flask, additional medium was added to reach a total volume of 4 ml and incubated in a carbon dioxide incubator (37 °C, 5% CO_2_). The medium was replaced every 3–4 days based on the rate of cell growth and the change in the color of the medium. To confirm the expression of GFP in BMSCs in vitro, we performed immunofluorescence staining using a GFP antibody (1:500, Santa Cruz Biotechnology), and cell nuclei were counterstained with DAPI (1:1000, Life Technologies). The GFP expression efficiency (%) was defined as the ratio of GFP-positive cells divided by the total number of cells (DAPI positive) per field. Five random fields per each well and four different wells at the same condition were evaluated to get the statistic value. Cell morphology was determined using a scanning electron microscope (SEM).

For GFP-BMSC transplantation treatment groups, cells were trypsinized with 0.05% trypsin solution for 3 min at 37 °C. After rinsing thrice, cells were used for transplantation. 1 × 10^5^ cells in 3 μL of DMEM medium were engrafted into the epicenter of the injury site at a delivery rate of 1 μL/min with a microinjection needle. The total number of cells for each treatment was the same. Animals in other groups received only saline injections.

### Enzyme-linked immunosorbent assay (ELISA)

To examine the inflammatory response at 24 h after MDL28170 treatment, brain tissue of the injected site was isolated and placed on ice. Each brain tissue was homogenized in RIPA lysis buffer (Thermo Fisher, USA) with the addition of protease inhibitors then centrifuged for 15 min at 12,000 rpm, 4 °C. The colorimetric ELISA kits were used to detect the cytokines (IL-1β, IL-6, TNF-α, IL-4, and IL-10) and transcription factor (NFκB) in the brain protein extract (R&D Systems, USA). For each ELISA analysis, 40 μL of sample was used without dilution in accordance with the manufacturer’s instructions.

### Survival assay of grafted cells

Rats were anesthetized with a lethal dose of chloral hydrate and transcardially perfused with 100 mL of saline followed by 100 mL of 4% paraformaldehyde (PFA) in 0.1 M PBS (pH 7.6). The tissue was fixed overnight in 4% PFA in 0.1 M PBS at 4 °C and cryoprotected in 30% sucrose for 36 h. Frozen sections of 10 μm thickness were prepared and fixed in 4% PFA for 20 min, washed with PBS (5 min each time for three times), then permeabilized with 0.3% Triton X-100 for 15 min, and washed with PBS (5 min each time for three times). The transplanted BMSCs can be detected directly with the 488 nm wavelength due to the transfection of GFP; cell nuclei were counterstained with DAPI. Samples were analyzed by fluorescence microscopy (BX51, Olympus, Japan). Five microscopic fields (× 40) from each section of each rat in each BMSC transplantation group were acquired to perform subsequent statistical analyses.

### Lesion volume assessment

Rats were sacrificed and transcardially perfused with saline and 4% PFA 7 days after cell transplantation. Sections were stained with Cresyl violet acetate, dehydrated, and mounted for analysis. The investigator measuring lesion area and contralateral hemisphere brain area using the NIH ImageJ program was blinded to the experimental conditions. Areas were multiplied by the distance between sections to obtain the respective volumes. Lesion volume was calculated as described previously [36]: (lesion volume/volume of contralateral hemisphere) × 100%.

### Western blot

At 30 min post TBI, 1.0 μl of 50 mM MDL28170 was injected into the center of the lesion site at a depth of 1.0 mm using a microinjection needle clamped by a stereotaxic instrument. Controls received an equal volume of the vehicle (20% DMSO, *v*/*v*). At 24 h after TBI, the consistent cortex tissue region of TBI area was separated. The routine detail procedures of western blot have been showed previously [37]. The following primary antibodies have been performed, including Bcl2 (Abacm, Rabbit, ab59348, 1:1000), Bax (Abacm, Rabbit, ab32503,1:1000), NFκB (Cell Signaling, Rabbit, #2144, 1:1000), p-IκB (Cell Signaling, Mouse, #2859, 1:1000), IκB (Cell Signaling, Rabbit, #4814, 1:1000), and α-tublin (Cell Signaling, Rabbit, #2144, 1:1000). For statistical analysis, each group contains three rats.

### Immunofluorescence staining

To explore the effect of MDL28170 on microgila activation, at 24 h after TBI, rats were sacrificed. The routine detail procedures for IbaI (Abcam, Goat, ab5076, 1:250) staining have been previously described [37]. For statistical analysis, four random images around TBI area were taken from each slide and each group contains four rats.

### Assessment of neurological function

Neurological function was assessed by a modified neurological severity score (mNSS) on the day before (baseline) and on days 7, 14, and 28 after transplantation by an investigator who was blinded to the experimental groups. The evaluations included motor, sensory, reflex, and balance tests. Neurological function was graded on a scale of 0–18 as previously described [38, 39]; the higher the score, the more severe the neurological impairment is. All rats were given enough time to become familiar with the testing environment before performing TBI, which was assessed by the rat’s ability to perform all the tests and a total mNSS (baseline) could be calculated.

### Statistical analysis

The data are presented as mean ± standard deviation. All values were analyzed using Prism software (GraphPad, USA). To compare differences between two groups, unpaired Student’s *t* test was used. For comparing differences involving three or more groups, one-way or two-way analysis of variance (ANOVA) was utilized. A *p* value of less than 0.05 or 0.01 or 0.001 is considered statistically significant.

## Results

### Characterization of cultured GFP-BMSCs

**Fig. 2:**
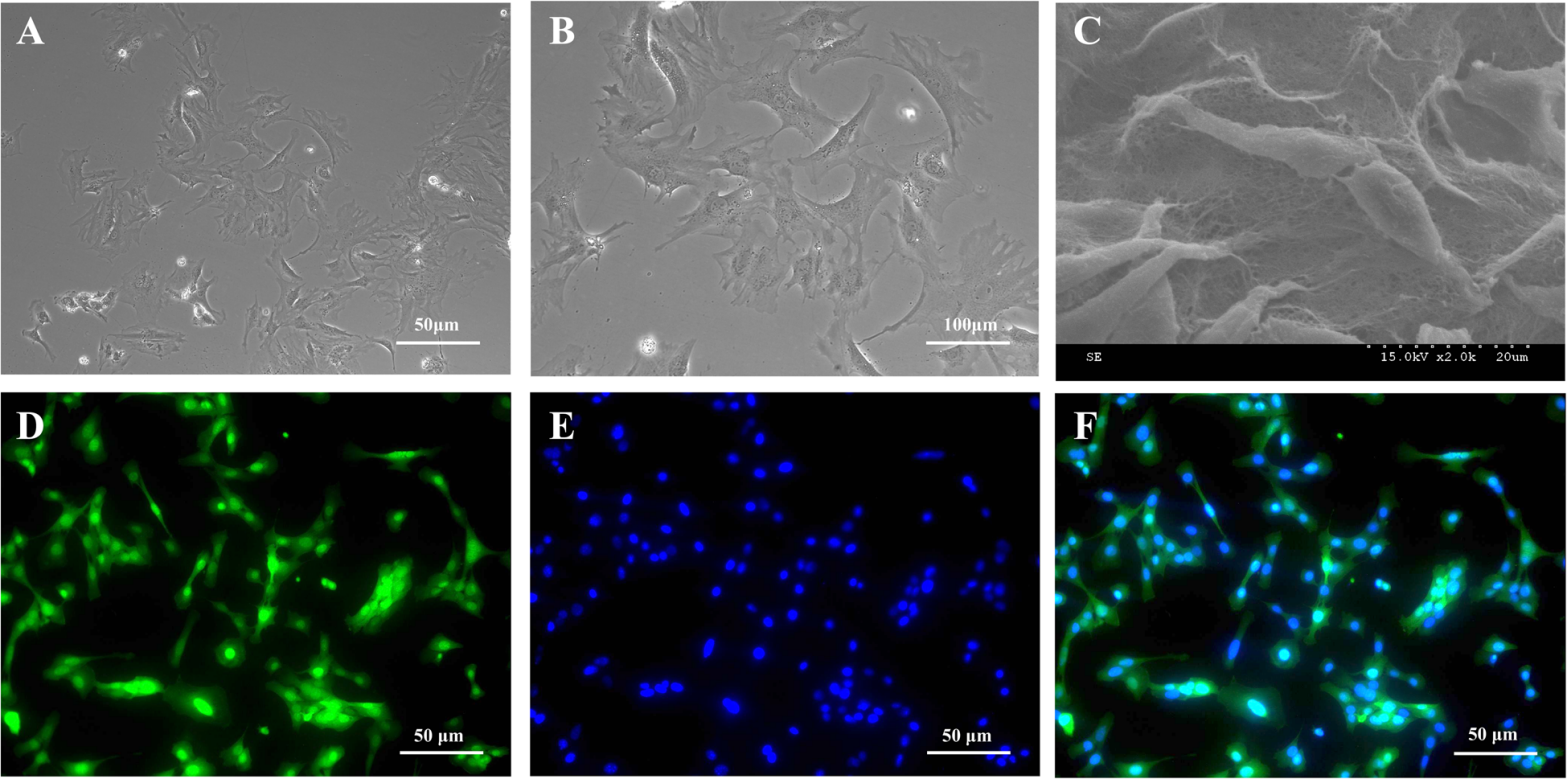
Characterization of BMSCs in vitro. **a**, **b** Representative images of BMSCs with phase-contrast view under the bright field. **c** SEM image showing the morphology of BMSCs in vitro*.* **d** BMSCs were labeled with GFP, emitting green fluorescence. **e** Nuclei, in blue, were counterstained with DAPI. **f** Merged image showing high GFP expression efficiency in BMSCs

Bright field images and SEM image show that BMSCs were long, rectangular cells adhering to the base of the culture flasks. To track the transplanted BMSCs, the cells were labeled with GFP, which emits a green fluorescence under the 488-nm wavelength. The result showed that about 94% BMSCs was labeled with GFP **(**Fig. 2**)**. Furthermore, CD44 marker was expressed exclusively in BMSC cell line in vitro **(**Additional file 1: Figure S1). The adipogenic commitment of BMSCs was evidenced by the ability of the cell to form mature lipid filled adipocytes **(**Additional file 1: Figure S1E).

### MDL28170 treatment in acute TBI phase decreased inflammatory effects

**Fig. 3:**
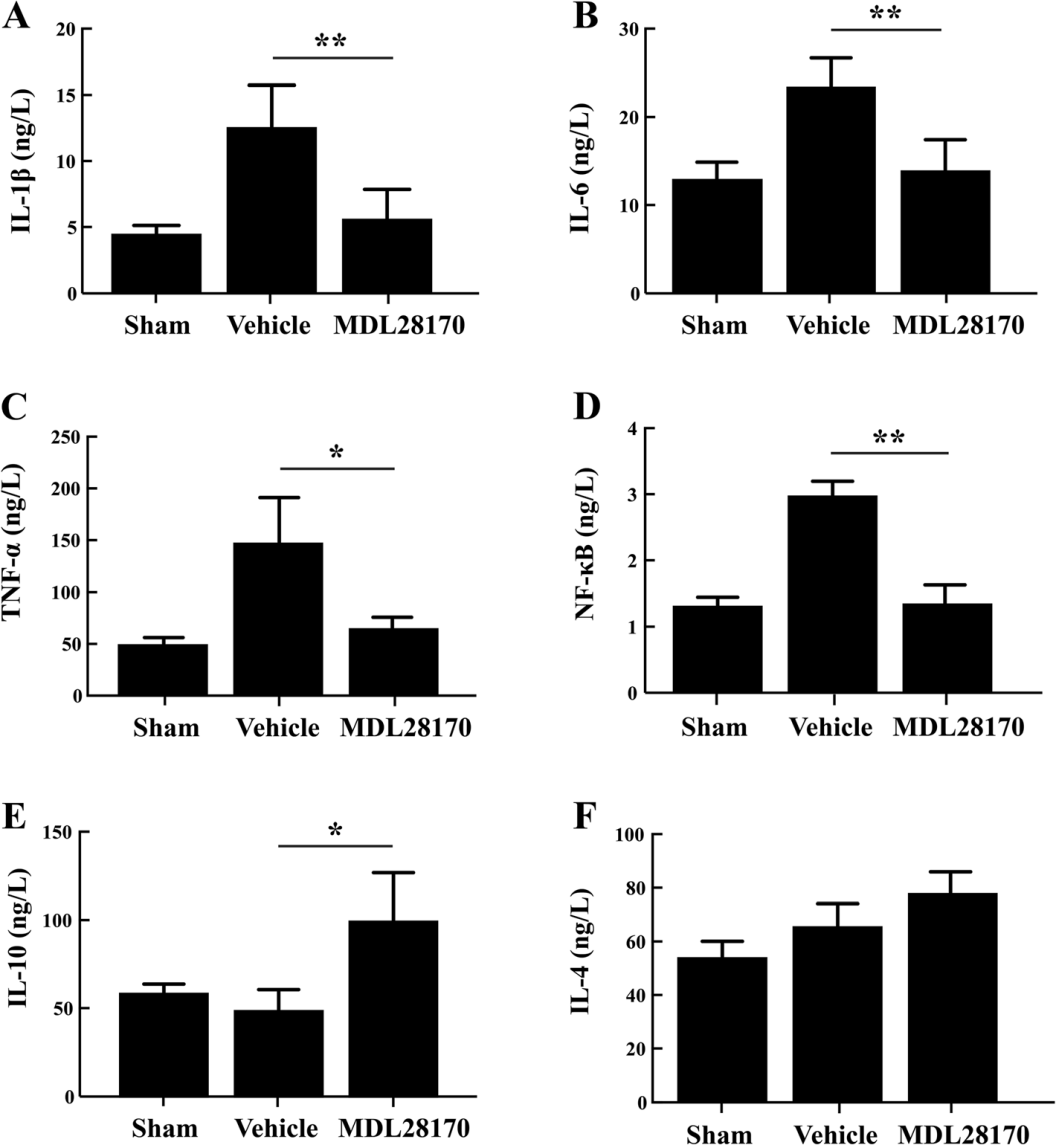
The effects of MDL28170 on the expression levels of pro-inflammatory and anti-inflammatory factors at 24 h after TBI. **a**–**d** Expression levels of pro-inflammatory factors IL-1β, IL-6, TNF-α, and inflammatory transcription factor NFκB decreased significantly after treatment with MDL28170 at 30 min after TBI compared with the vehicle group, respectively. **e**, **f** Increased expression levels of anti-inflammatory factors IL-10 and IL-4, respectively. *P < 0.05, **P < 0.01 by one-way ANOVA followed by Turkey post-tests (*n* = 4). TBI, traumatic brain injury

To check the efficacy of MDL28170 as a calpain inhibitor, BMSCs were treated with MDL28170 or vehicle (0.5% DMSO); at 24 h later, cell samples were collected and the Capn1 gene expression level was determined by qPCR assay, in which we found that Capn1 expression was significantly decreased in the MDL28170 treatment group compared with vehicle treatment group (Additional file 1: Figure S2), suggesting the calpain inhibition effect of MDL28170. In addition, we assayed the levels of pro-inflammatory factors (IL-1β, IL-6, TNF-α) and inflammatory transcription factor (NFκB), as well as anti-inflammatory factors, including IL-10 and IL-4, at the site of injury to determine the extent of cytokine activation **(**Fig. 3**)**. Treatment with MDL28170 markedly reduced the levels of all pro-inflammatory cytokines at 24 h after TBI. On the other hand, MDL28170 treatment increased the levels of both anti-inflammatory cytokines IL-4 and IL-10, with a significant effect being observed for IL-10 only (*P* < 0.05) at 24 h after TBI compared with the vehicle group. These data imply that MDL28170 inhibits pro-inflammatory effects induced by TBI and promotes certain anti-inflammatory effects.

### MDL28170 enhanced the survival ratio of grafted cells in host tissue

**Fig. 4:**
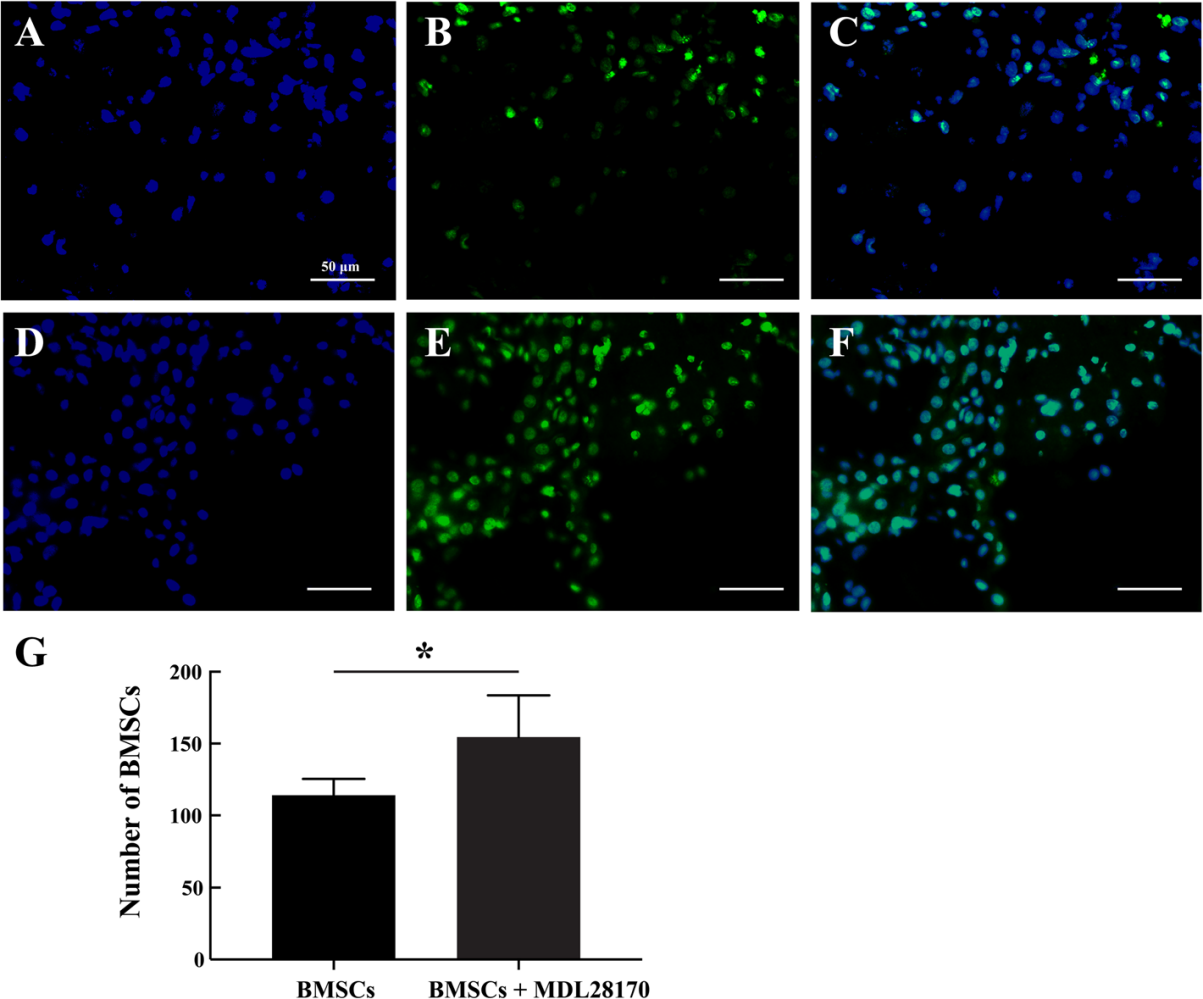
Survival and integration of transplanted cells in vivo. **a**–**c** BMSCs alone group: nuclei, in blue, were counterstained with DAPI; transplanted BMSCs were labeled with GFP, which emit a green fluorescence; grafted BMSCs can survive in host tissue. **d**–**f** MDL28170+GFP-BMSC transplantation group: transplanted BMSCs were able to survive better with MDL28170 pretreatment. **g** Quantification of the number of BMSCs per field in TBI rats pretreated with MDL28170 at 7 days after transplantation. Quantified summary shows the increased number of BMSCs surviving in the MDL28170 pretreated group, *P < 0.05 by two-tailed Student’s *t* test (*n* = 5). BMSCs, bone marrow mesenchymal stem cells

Cells emitting the green fluorescence were found in the precontusional tissue, confirming these as transplanted BMSCs and demonstrating that grafted cells could survive and migrate around the injury site. Furthermore, compared with BMSCs implanted alone, the number of surviving BMSCs in the MDL28170 pretreatment group was significantly increased at 7 days after transplantation (*P* < 0.05; Fig. 4e). This finding illustrates that the calpain inhibitor, MDL28170, plays an important role in enhancing the survival of transplanted BMSCs. Meanwhile, among the survival cells, the majority of grafted GFP-BMSCs were co-immunostained with Ki67 at the MDL28170-preconditioned lesion site, indicating that with the pretreatment of MDL28170, a large amount of GFP-BMSCs were not only able to survive, but also maintain the cell proliferation ability **(**Additional file 1: Figure S3).

### MDL28170 reduced lesion volume after transplantation of BMSCs in TBI

**Fig. 5:**
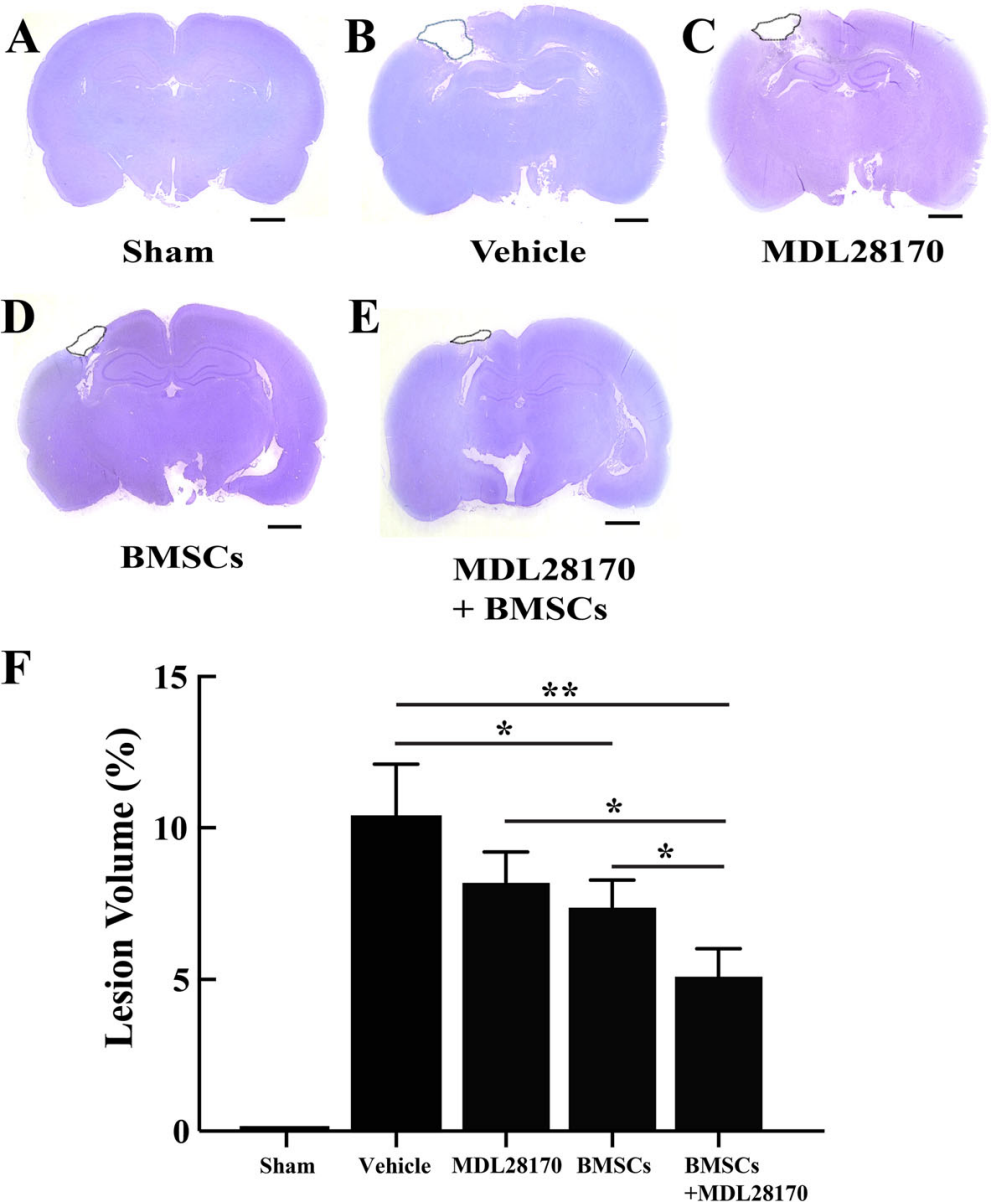
Lesion volume assessment of TBI brain sections stained with Cresyl violet 7 days after treatment or cell transplantation. **a** Sham group, no injury. **b** TBI with vehicle (20% DMSO, *v*/*v*). **c** TBI with MDL28170 treatment. **d** TBI with BMSC transplantation. **e** TBI with MDL28170 pretreatment then BMSC transplantation. **f** Quantification of lesion volume in each group (*n* = 3 for the sham group, *n* = 5 for all other groups). *P < 0.05, **P < 0.01 by one-way ANOVA followed by Turkey post-tests. Scale bars, 2 mm (**a**–**e**). TBI, traumatic brain injury; BMSCs, bone marrow mesenchymal stem cells

Since MDL28170 treatment promoted anti-inflammatory function and enhanced BMSC survival, we further examined whether these two favorable conditions could alleviate parenchymal tissue loss after TBI. Therefore, we measured TBI-induced lesion volume after transplantation using Cresyl violet-stained coronal brain sections at 7 days after injury. Representative images from each group are shown in Fig. 5a–e. BMSC transplantation significantly reduced TBI-induced lesion volumes compared with the vehicle-treated group. However, there is no significant decrease of lesion cavity in the MDL28170-only treatment group compared with the vehicle. Interestingly, pretreatment with MDL28170 followed by BMSC transplantation significantly decreased lesion volume compared with BMSCs or MDL28170 only treated groups at 7 days after TBI (Fig. 5f). These data, together with data shown in Figs. 3 and 4, indicate that the calpain inhibitor, MDL28170, exerts its neuroprotective effect by inhibiting pro-inflammatory processes to provide BMSCs with a favorable microenvironment for survival and tissue regeneration.

### Assessment of neurological function after BMSC transplantation

**Fig. 6:**
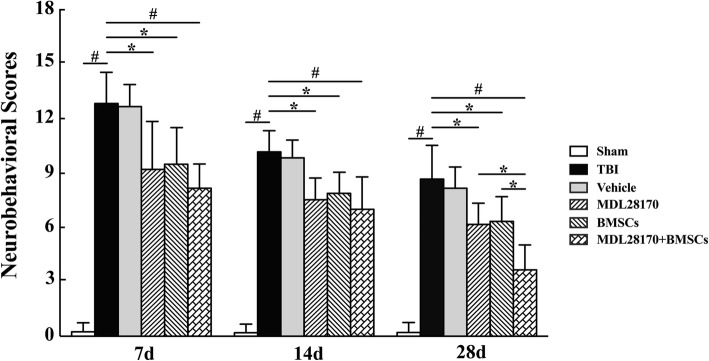
Functional assessment of neurological behavior after TBI. mNSS tests, 7, 14, and 28 days after TBI surgery exhibited that the scores significantly increased immediately after TBI (*P* < 0.01 versus sham). However, compared with the TBI group, 7 and 14 days after the injury, the mNSS scores of rats that received the treatment of BMSCs or MDL28170 alone were significantly decreased (*P* < 0.05), and the scores in co-grafted rats are even lower (*P* < 0.01). On 28 days after injury, combination therapy of BMSCs and MDL28170 achieved a significant reduction of mNSS scores compared to single-treatment group. Data are analyzed using two-way ANOVA followed by Turkey post-tests at each time point, *n* = 6 per group. mNSS, modified neurological severity score; BMSCs, bone marrow-derived mesenchymal stem cells; TBI, traumatic brain injury

Before TBI or sham operation (i.e., at baseline, 1 day before operation), rats present with a score of 0 by mNSS evaluation and showed normal brain function. Then, mNSS tests were performed on 7, 14, and 28 days post TBI showing impairment of locomotor functions. On 7 and 14 days after the injury, the mNSS of rats that received BMSCs only or BMSCs with MDL28170 significantly decreased (*P* < 0.05 and *P* < 0.01, respectively). At 28 days after injury, transplantation of BMSCs with MDL28170 treatment achieved a significant reduction of mNSS score compared to MDL28170 or BMSCs alone, indicating that BMSC transplantation with calpain inhibitor pretreatment can achieve a better improvement of neurological function at 4 weeks after injury compared to BMSC transplantation only (Fig. 6).

### MDL28170 reduced cell apoptosis and inhibited NFκb-Iκb signaling pathway after TBI

**Fig. 7:**
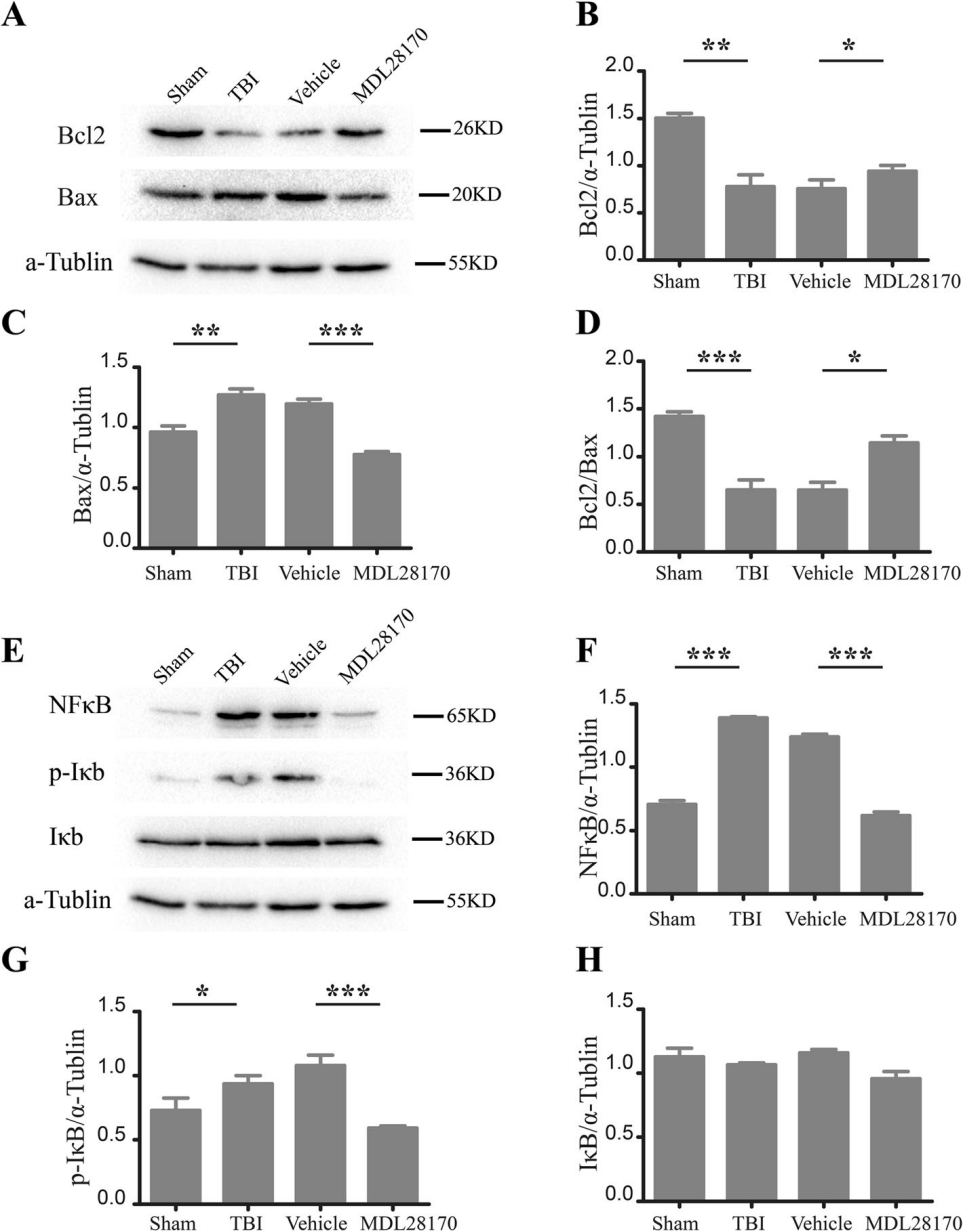
MDL28170 reduces cell apoptosis and inhibited NFκb-Iκb pathway signaling after TBI. **a** Representative western blot images for the protein levels of Bcl2, Bax, and α-tublin. **b**–**d** Quantification of Bcl2 and Bax protein expression levels (*n* = 3 per group; *p < 0.05, **p < 0.01, ***p < 0.001 by one-way ANOVA followed by Turkey post-tests). **e** Representative western blot images for the protein levels of NFkb, p-Ikb, Ikb, and α-tublin. **f**–**h** Quantification of NFkb, p-Ikb, and Ikb protein expression levels (*n* = 3 per group; *p < 0.05, ***p < 0.001 by one-way ANOVA followed by Turkey post-tests)

With the preconditioning of MDL28170 after TBI, the inflammation level at brain lesion site was significantly attenuated (Fig. 3), along with an enhanced survival ratio of implanted GFP-BMSCs (Fig. 4). To investigate the underlying protective mechanisms mediated by MDL28170 treatment, the grafted cells’ apoptosis condition and the NFκB-Ikb signaling pathway activity were explored by western blot. Compared with vehicle treatment group, we found that the protein level of Bcl2 was significantly increased in the MDL28170 treatment group, while the protein level of Bax was dramatically decreased (Fig. 7a–c). Furthermore, the increased value of Bcl2/Bax in MDL28170 treatment group also indicated that MDL28170 could reduce cell apoptosis (Fig. 7d).

Previously, we have showed that the expression of NFκb after TBI was downregulated by MDL28170 treatment in the ELISA assay (Fig. 3d). This data agrees well with the western blot results, which also support that the protein level of NFκB was decreased after MDL28170 treatment (Fig. 7e, f). As we know, Iκb and p-Iκb are the downstream biomarkers of NFκB, and NFκB can mediate the phosphorylation of Iκb. Interestingly, our data showed that MDL28170 decreased the protein level of p-Iκb; however, no significant effect of protein level of Iκb was observed here (Fig. 7e, g, h). Put together, the results demonstrated that the administration of MDL28170 after TBI could inhibit cell apoptosis and reduce inflammation level by inhibit NFκB-Iκb signaling pathway.

### MDL28170 administration inhibited microglia activation after TBI

**Fig. 8:**
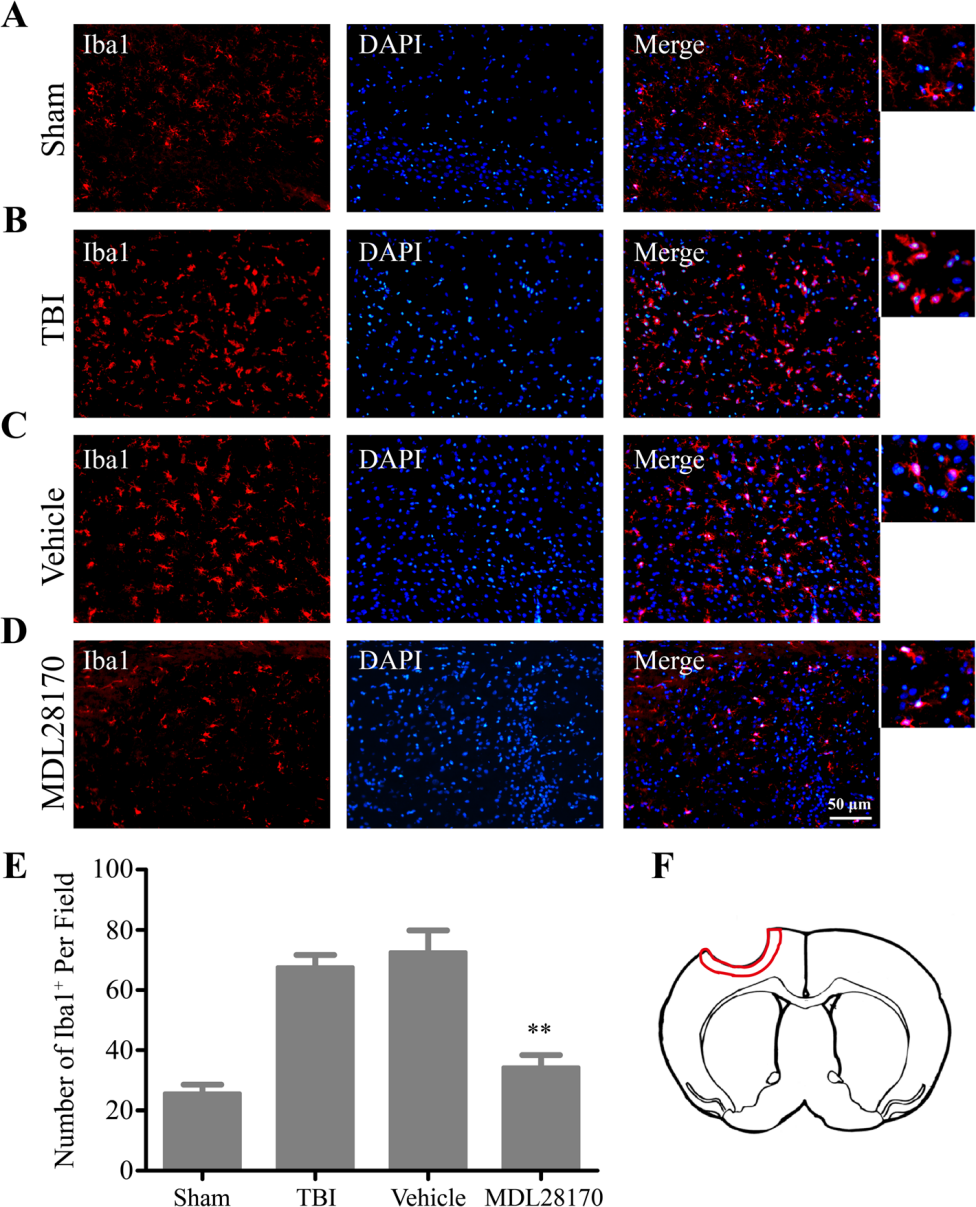
MDL28170 inhibited the microglia activation after TBI. **a**–**d** Representative images of Iba1 staining in different groups (Sham, TBI, TBI+Vehicle, TBI+MDL28170). **e** Quantification of Iba1-positive cell number per field (*n* = 4 per group; **p < 0.01 by one-way ANOVA followed by Turkey post-tests). **f** The red cycle indicates the area where the images were taken

Microglia as the major innate immunity cell type in the brain plays a critical role in regulating the inflammation response after TBI. Even though studies have been showed that MDL28170 could cause the reduction of inflammation level after TBI, the cellular level mechanism has not been well investigated. To explore the effect of MDL28170 on the activation of microglia, Iba1 as a microglia biomarker was stained in different groups: sham, TBI, vehicle, and TBI+MDL28170 groups. The images were taken at the lesion site, indicated in Fig. 8e. After the formation of TBI, the Iba1-positive cells at the lesion site were dramatically increased. More importantly, we found that with the administration of MDL28170, the number of Iba1-positive cells was significantly diminished (Fig. 8a–d), suggesting that MDL28170 as a calpain inhibitor could alleviate the microglia activation at the lesion site of brain after TBI.

## Discussion

In this study, our results demonstrate for the first time that the calpain inhibitor, MDL28170, administered by intracranial microinjection shortly following injury can not only attenuate the effects of an inflammatory microenvironment, but also enhance the survival rate of BMSCs at the contusive site, decrease lesion volume, and improve functional outcome. Taken together, our results provide preclinical experimental evidence for the efficacy of combinatorial therapy with MDL28170 and BMSCs to aid in functional recovery after a brain injury.

The effects of acute TBI include a complex cascade of pathophysiological sequelae such as excitotoxicity, generation of free radicals (elevated levels of reactive oxygen species and reactive nitric oxide), release of inflammatory molecules, and diffuse axonal and neuronal injury [40, 41]. Inflammatory responses are reported to be a crucial mechanism in secondary injury after TBI. Early responses of the inflammatory reactive cells result in a conspicuous accumulation of other inflammatory mediators such as cytokines and adhesion molecules [42, 43]. The massive death of donor cells in the contusion area during the acute phase resulting from increased free radicals and inflammatory responses immensely lowers the efficacy of the cell-based treatment. In order to improve the effect of stem cell-based therapy, various strategies have been adopted to develop and optimize the protocols to enhance donor stem cell survival post-transplantation, with special attention being paid to preconditioning approaches [44, 45]. Presently, several preconditioning triggers are being tested in stem cell-based therapy and have shown to increase the tolerance of transplanted cells to multiple injurious insults [46, 47].

An increasing number of studies suggest that calpains could participate in acute and chronic inflammatory processes under pathological conditions by acting as inflammatory regulators. For example, treatment with calpain inhibitor can reduce calpain activity in immune cells in the periphery to potentially block T cell activity and immune cell migration [48]. In accordance with the literature, our study also showed that MDL28170 as a calpain inhibitor could alleviate the microglia activation at the lesion site of brain after TBI (Fig. 8). As reported recently, an increased calpain activity also correlates with greater production of pro-inflammatory IL-2/IFN-γ cytokines and decreased levels of anti-inflammatory cytokines IL-10 and IL-4, suggesting that calpain plays a modulatory role in T cell activation and production of Th1/Th2 type cytokines during the relapsing and remitting phase of some diseases [37, 49]. Moreover, it has been shown that calpain inhibitors can reduce TNF-α mRNA expression [50, 51] and proteasomal degradation of IκB and hence inhibit NFκB-driven transcription of pro-inflammatory cytokines and chemotactic factors [52]. Meanwhile, inhibiting calpain by overexpressing a minimal domain of calpastatin could also coordinately suppress IL-1β and IL-6 activities [53, 54]. In line with these studies, we have shown here that inhibition of calpain by calpain inhibitor, MDL28170, reduced the levels of pro-inflammatory cytokines (TNF-α, IL-1β, IL-6) and inflammatory transcription factor (NFκB) after TBI, but increased the levels of anti-inflammatory factors IL-10 and IL-4. The neuroprotective microenvironment attributed to the pretreatment with MDL28170, 30 min after TBI and before BMSC transplantation, may be of benefit to enhance the survivability of transplanted cells. Calpain inhibitors have been reported to inhibit both apoptosis and necrosis [55, 56], have neuroprotective effects in numerous rodent neurotrauma models, including TBI, spinal cord injury [23], and focal cerebral ischemia [45, 56, 57]. In fact, treatment with MDL28170 rescued transplanted BMSCs in the injured spinal cord by modulating ER stress-induced apoptosis [58]. MDL28170 also enhanced the survival of transplanted Schwann Cells 7 days after transplantation into the contused spinal cord [22]. Similarly, we demonstrated that MDL28170 pretreatment could reduce cell apoptosis and significantly enhanced the survivability of transplanted BMSCs after TBI compared with the BMSCs-only group. Therefore, these results support the use of calpain inhibitors as a promising new treatment for promoting the survival of transplanted cells.

The fact that a reduction in brain damage after TBI has been shown via BMSC transplantation alone [59, 60] corroborates with our data from this study. However, there is no significant decrease of lesion cavity in the MDL28170-only treatment group compared with the TBI group. This lack of effect on lesion volume has been seen with other calpain inhibitors, suggesting that pharmacological calpain inhibition alone though able to reduce axonal injury, may not in fact produce a measurable reduction in lesion volume [52, 61]. To the best of our knowledge, the combinatorial effects of MDL28170 and transplantation of BMSCs have not been investigated. Here, we showed that the pretreatment of MDL28170 followed by BMSC transplantation could achieve at least a 30% improvement in lesion volume compared with the BMSCs-only or MDL28170-only groups at 7 days after TBI. This may be due to the enhanced survival ratio of transplanted BMSCs and the neuroprotective effect exerted by MDL28170. Previous studies have also shown that MDL28170 was able to reduce motor neuron death and improve locomotor function [20]. We demonstrated that the combination of MDL28170 and transplanted BMSCs saw a more distinct recovery of neurological function versus transplanted BMSCs alone, especially in the long-term study, which may be attributable to the anti-neurodegeneration and anti-inflammatory effects of the calpain inhibitor MDL28170. Taken together, our present work strongly suggests that the combination of calpain inhibitor pretreatment followed by cell transplantation produces more robust neuroprotective and functional recovery effects than either agent used alone and therefore warrants further study. For instance, to further elucidate the neuroprotective mechanism of the calpain inhibitor MDL28170, long-term experiments aiming to observe the number, localization, and differentiation status of transplanted cells in the lesioned brain are needed. Also, to study the mechanism of functional brain recovery more in-depth, we would suggest examining the regulation of neurotrophic factors, possible axonal regeneration and angiogenesis, and the potential formation of networks between endogenous neurons and transplanted stem cells differentiated neurons. Lastly, additional observations involving larger cohorts are required soon, with more definite conclusions regarding the safety of stem cell treatment to be made.

## Conclusion

This study is the first to evaluate the use of MDL28170 combined with BMSC transplantation after TBI. Our data suggest that a single dose of MDL28170 in the acute phase of TBI improves the microenvironment by inhibiting inflammatory processes, which facilitated the survival of grafted BMSCs, leading to the reduction of lesion volume and the improvement of neurological function. Thus, we suggest a novel therapeutic strategy for TBI treatment by using a combination of MDL28170 and BMSCs. This promising new approach for promoting the survival of transplanted stem cells may be immensely beneficial for TBI patients relying on cell-based regenerative medicine.

## Additional file

Additional file 1: **Figure S1.** Characterization of GFP-BMSCs in vitro. (A, B, C) Representative images of CD44 biomarker staining at 7, 14, and 21 days with or without GFP over expression. (D) Representative images of CD34 biomarker staining. (E) Representative images of GFP-BMSCs under bright field. (F) Adipogenic differentiation potential of BMSC: generation of lipid droplets under adipogenic-induction medium for 16 days. The red arrows indicate lipid droplets. **Figure S2.** MDL28170 inhibits Capn 1 gene expression. qRT-PCR analysis for Capn 1 gene expression level. (*n* = 3 per group; **p < 0.01, ***p < 0.001 by one-way ANOVA followed by Turkey post-tests). **Figure S3.** Proliferation state of grafted GFP-BMSCs at the lesion site of TBI brain. (A) Grafted GFP-BMSCs co-immunostained with Ki67 in the MDL28170-preconditioned TBI rats at 7 days post transplantation. (B) Quantification of Ki67^+^GFP^+^/GFP^+^ ratio of implanted GFP-BMSCs at the lesion site where pretreated with MDL28170 (*n* = 4). (PDF 7048 kb)

## Abbreviations

ANOVA: Analysis of variance
BBB: Blood-brain barrier
BMSCs: Bone marrow-derived mesenchymal stem cells
DMSO: Dimethylsulfoxide
ELISA: Enzyme-linked immunosorbent assay
GFP: Green fluorescent protein
mNSS: Modified neurological severity score
PFA: Paraformaldehyde
SD: Sprague-Dawley
SEM: Scanning electron microscope
TBI: Traumatic brain injury
