# Supplementary material for: Calpain inhibitor MDL28170 improves the transplantation-mediated therapeutic effect of bone marrow-derived mesenchymal stem cells following traumatic brain injury
Source: Stem Cell Res Ther. 2019 Mar 15;10:96. doi: 10.1186/s13287-019-1210-4 (PMC6420775; doi:10.1186/s13287-019-1210-4)
Supplement: Supplementary file 1 — Figure S1. Characterization of GFP-BMSCs in vitro. (A, B, C) Representative images of CD44 biomarker staining at 7, 14, and 21 days with or without GFP over expression. (D) Representative images of CD34 biomarker staining. (E) Representative images of GFP-BMSCs under bright field. (F) Adipogenic differentiation potential of BMSC: generation of lipid droplets under adipogenic-induction medium for 16 days. The red arrows indicate lipid droplets. Figure S2. MDL28170 inhibits Capn 1 gene expression. qRT-PCR analysis for Capn 1 gene expression level. (n = 3 per group; **p < 0.01, ***p < 0.001 by one-way ANOVA followed by Turkey post-tests). Figure S3. Proliferation state of grafted GFP-BMSCs at the lesion site of TBI brain. (A) Grafted GFP-BMSCs co-immunostained with Ki67 in the MDL28170-preconditioned TBI rats at 7 days post transplantation. (B) Quantification of Ki67+GFP+/GFP+ ratio of implanted GFP-BMSCs at the lesion site where pretreated with MDL28170 (n = 4). (PDF 7048 kb) [file 13287_2019_1210_MOESM1_ESM.pdf]

Calpain inhibitor MDL28170 improves the transplantation-mediated therapeutic effect of bone marrow-derived mesenchymal stem cells following traumatic brain injury

Jiangnan Hu[1, 2], Lefu Chen[1], Xujun Huang[3], Ke Wu[1], Saidan Ding[1], Weikan Wang[2], Brian Wang[2], Charity Smith[2], Changhong Ren[4], Haoqi Ni[1], Qichuan ZhuGe[1*] and Jianjing Yang[1*]*

[1] *Zhejiang Provincial Key Laboratory of Aging and Neurological Disorder Research, The First Affiliated Hospital of Wenzhou Medical University, Wenzhou 325000, China*

[2] *Department of Pharmaceutical Sciences, University of North Texas Health Science Center, Fort Worth, Texas 76107, USA*

[3]*Department of Intensive Care Unit (ICU), Hengdian Wenrong Hospital, Jinhua, 322100, China.*

[4]*Beijing Key Laboratory of Hypoxic Conditioning Translational Medicine, Xuanwu Hospital, Capital Medical University, Beijing, China.*

***Corresponding Author:**

Jiangnan Hu, M.D., Department of Pharmaceutical Sciences, University of North Texas Health Science Center, Fort Worth, Texas 76107, USA, Tel: 817-735-0441, Fax: 817-735-2603. E-mail: hu.jiangnan@hotmail.com

Zhuge Qichuan, M.D., Zhejiang Provincial Key Laboratory of Aging and Neurological Disorder Research, The First Affiliated Hospital of Wenzhou Medical University, Wenzhou, Zhejiang, 325000, China, Tel: 8655755578085; Fax: 86 577 88069607; Email: zhugeqichuan@vip.163.com

Jianjing Yang, M.D., Ph.D. Zhejiang Provincial Key Laboratory of Aging and Neurological Disorder Research, The First Affiliated Hospital of Wenzhou Medical University, Wenzhou, Zhejiang, 325000, China, Tel: 8655755578085; Fax: 86 577 88069607; Email: yangjianjing2@163.com

Part S1 Identification of osteoblastic and adipogenic properties of GFP-BMSCs

BMSCs were transduced with plasmids which contain GFP expression domain. When BMSCs grew into 90% confluence, cells were passaged. To test the immune-phenotype potential of BMSCs, GFP-BMSCs were stained with CD44 antibody (Abcam, Rabbit, ab157107) at days 7, 14 and 21. We found that CD44 marker was expressed exclusively in BMSCs cell line **(Figure S1A-C)**, moreover, we confirmed that CD34 (Abcam, Rabbit, ab81289) was not expressed in BMSCs cell line **(Figure S1D).** To test the adipocyte differentiation potential of GFP-BMSCs, cells were cultured for 16 days with adipocyte medium: IBMX (Sigma, I5879, 0.5μmol/L), DEXD (Sigma, 4902, 1μmol/L), FBS (Thermo, 10099141, 10%), DMEM (Thermo, SH30243.01), Penicilillin-Streptomycin (Thermo, 15130122, 1%). The media was changed every three days. The adipogenic commitment of BMSCs were evidenced by the ability of the cell to form mature lipid filled adipocytes **(Figure S1F)**.

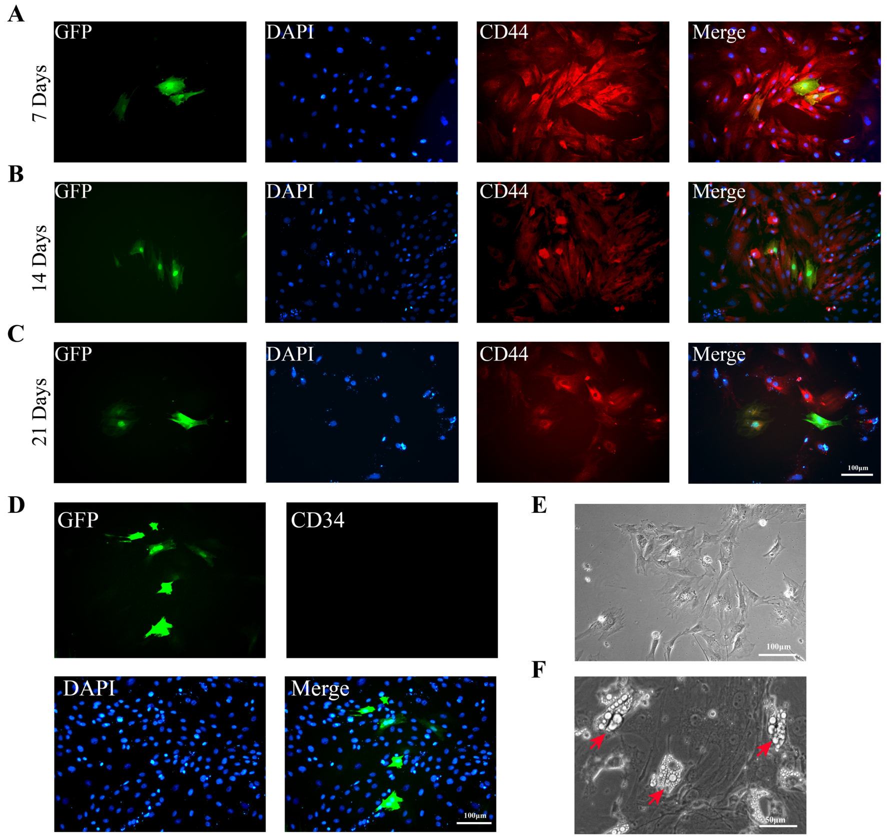

Figure S1. Characterization of GFP-BMSCs *in vitro*. (A, B, C) Representative images of CD44 biomarker staining at 7, 14, 21 days with or without GFP over expression. **(D)** Representative images of CD34 biomarker staining. **(E)** Representative images of GFP-BMSCs under bright field. **(F)** Adipogenic differentiation potential of BMSC: generation of lipid droplets under adipogenic-induction medium for 16 days. The red arrows indicate lipid droplets.

Part S2 Calpain inhibition effect of MDL28170 *in vitro*

To determine the inhibition effect of MDL28170 on Capn1 gene expression, BMSCs were treated with 10 μM MDL28170, 20 μM MDL28170 or vehicle (0.5 % DMSO). After 24 hours, BMSCs were collected and the RNA sample was isolated by routine procedures. Reverse transcription (Thermo, K1622) and qPCR (Roche, 04913922001) were performed according to standard procedure of the kits. The detail primer sequences were listed: Capn1: Forward 5'AACTCCCCTTCCCCAGGATG; Reverse 5'CTGGCCCAGGTACTTGATGG; GAPDH: Forward 5'AGTGCCAGCCTCGTCTCATA; Reverse5'ATGAAGGGGTCGTTGATGGC. As shown in **Figure S2**, the expression level of Capn1 was significantly decreased in the MDL28170 treatment groups at the concentration of 10 μM and 20 μM.

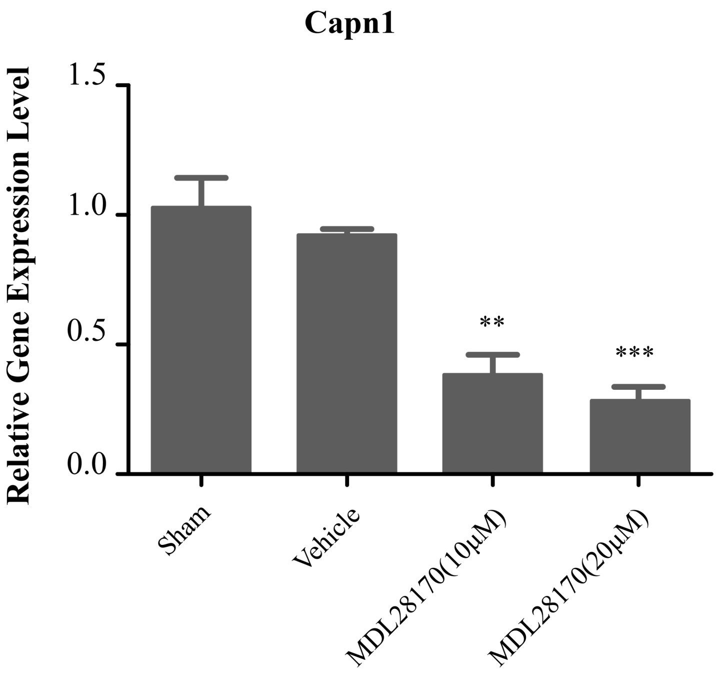

Figure S2. MDL28170 inhibits Capn 1 gene expression. qRT-PCR analysis for Capn 1 gene expression level. (n=3 per group; **p < 0.01, ***p < 0.001 by One-Way ANOVA followed by Turkey post-tests).

Part S3 Proliferation state of grafted GFP-BMSCs at the lesion site of TBI brain

To analysis the proliferation state of GFP-BMSC after injection, 7 days after injection and MDL28170 administration, rats were sacrificed and stained with Ki67(Abcam, Rabbit, ab15580, 1:500). We found that the majority of grafted GFP-BMSCs were co-immunostained with Ki67 at the MDL28170-preconditioned lesion site, indicating that with pretreatment of MDL28170, a large amount of GFP-BMSCs were not only able to survive, but also maintain the cell proliferation ability **(Figure S3)**.

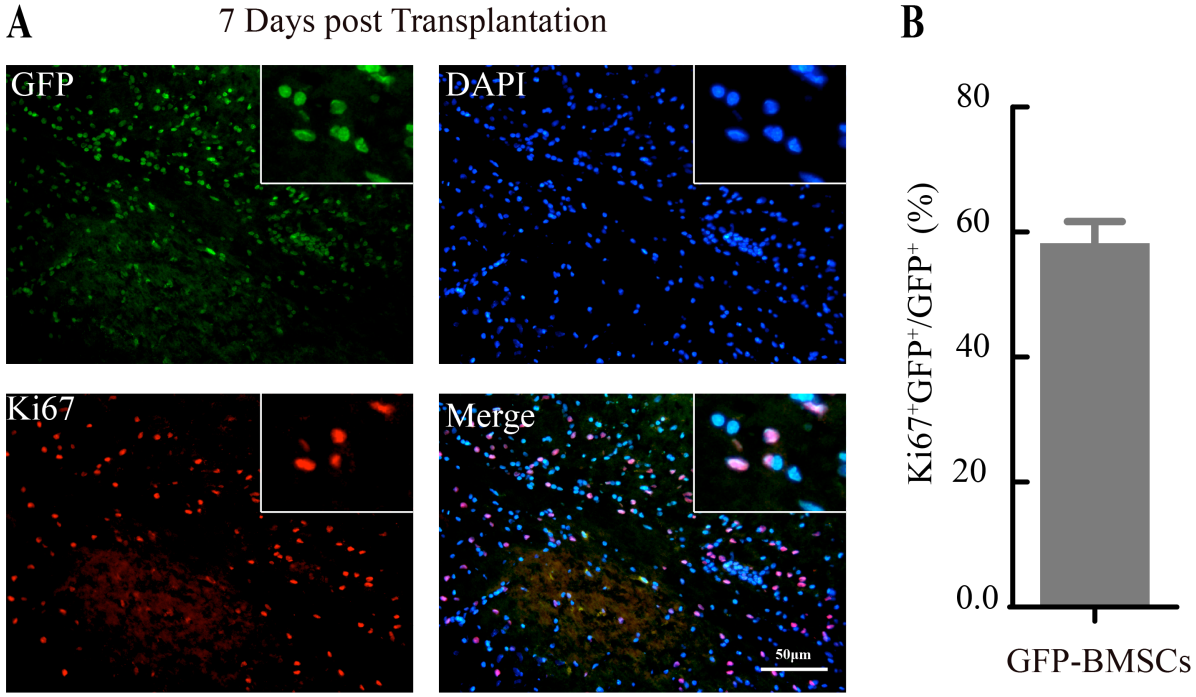

Figure S3. Proliferation state of grafted GFP-BMSCs at the lesion site of TBI brain. (A) Grafted GFP-BMSCs co-immunostained with Ki67 in the MDL28170-preconditioned TBI rats at 7 days post transplantation. **(B)** Quantification of $Ki67^+GFP^+/GFP^+$ ratio of implanted GFP-BMSCs at the lesion site where pretreated with MDL28170 (n = 4).
